# Awareness, Attitudes, and Perceptions Toward Deceased Organ Donation Among Healthcare Students and Professionals: A Mixed-Methods Study

**DOI:** 10.7759/cureus.92994

**Published:** 2025-09-23

**Authors:** Prasad VG, Anaswara MB, Mamatha Jayachandran, Ebin V Abraham, Subramania Iyer

**Affiliations:** 1 Centre for Allied Health Sciences, Amrita Vishwa Vidyapeetham - Amrita Institute of Medical Sciences, Kochi, IND; 2 Neurology, Amrita Vishwa Vidyapeetham - Amrita Institute of Medical Sciences, Kochi, IND; 3 Pharmacology, Amrita Vishwa Vidyapeetham - Amrita Institute of Medical Sciences, Ernakulam, IND; 4 Plastic and Reconstructive Surgery, Head and Neck Surgery, Amrita Vishwa Vidyapeetham - Amrita Institute of Medical Sciences, Kochi, IND

**Keywords:** awareness and attitudes, deceased donor, organ donation, perception, transplantation

## Abstract

Background

Deceased organ donation is essential for addressing organ shortages and meeting the increasing demand for transplantation. With thousands of patients worldwide awaiting transplants, organ donation not only provides a second chance for recipients but also reduces the burden of long-term treatment costs, such as dialysis. Healthcare professionals play a vital role in promoting donation, and their awareness, attitude, and perception significantly influence this process. This study aimed to evaluate the awareness, attitude, and perception of medical and allied health students, as well as junior and senior medical staff, toward deceased organ donation.

Methods

A mixed-method study, comprising descriptive analysis of quantitative data and thematic analysis of qualitative data, was conducted. For the quantitative component, 45 medical students, 35 Allied Health Sciences (AHS) students, 63 junior doctors, and 21 senior doctors from a tertiary care university teaching hospital were assessed to determine their awareness and perceptions using a validated questionnaire. The questionnaire included both closed-ended and open-ended questions. For the qualitative component, two to three participants from each group were selected for in-depth interviews to explore their perceptions of deceased organ donation. Quantitative data were analyzed descriptively, whereas qualitative data were analyzed thematically. Written informed consent was obtained before data collection using Google Forms.

Results

While 99.3% of participants were aware of organ donation, knowledge gaps persisted, particularly regarding brain death. Notably, 11.4% of AHS students and 9.5% of junior doctors incorrectly believed that brain death is reversible. Awareness of organ donation laws was highest among senior doctors (85.7%) and lowest among AHS students (20%). Despite a high willingness to donate (84.1%), actual donor registration remained low (13.4%), highlighting procedural and informational barriers. Family influence, cultural beliefs, fear of organ trafficking, and lack of knowledge regarding the registration process emerged as key barriers. Facilitators included enhanced engagement of healthcare professionals, implementing educational initiatives, expansion of government policies, and targeted media campaigns to increase donor registrations and public trust.

Conclusion

The study underscores the need for targeted educational interventions to address knowledge gaps, particularly regarding brain death and legal aspects of organ donation. Addressing cultural concerns, fostering family discussions, and increasing transparency in organ allocation are crucial for improving donor participation. Strengthening the role of healthcare professionals as advocates, integrating structured discussions on organ donation into medical education, and leveraging digital media for awareness campaigns can significantly enhance organ donation rates and help meet the growing demand for transplantation.

## Introduction

Organ donation is a life-saving intervention that provides hope for patients suffering from organ failure due to disease, injury, or genetic conditions. A single deceased donor can save multiple lives by donating vital organs, including the heart, kidneys, liver, and lungs, as well as tissues such as corneas and skin. Living donors can also contribute by donating a kidney or a portion of their liver [1]. By choosing to become organ donors, individuals make a lasting impact on public health. The primary challenge in global organ transplantation efforts is the persistent shortage of organs [2]. Despite advances in medical science, the gap between organ supply and demand remains a major challenge worldwide, particularly in countries such as India, where organ donation rates are low [3]. Increasing the number of transplants from deceased donors is essential to address this scarcity. The Madrid Resolution, developed during the Third Global Consultation on Organ Donation and Transplantation, represents a major collaborative effort among the World Health Organization (WHO), the Spanish National Transplant Organization (ONT), and The Transplantation Society (TTS). It emphasizes that organ donation and transplantation are national responsibilities, requiring comprehensive strategies to meet patient needs [4,5]. The resolution advocates for self-sufficiency, ethical practices, and robust national frameworks to enhance organ donation and transplantation worldwide while preventing exploitative practices. Deceased organ donation is central to achieving self-sufficiency; however, in India, living organ donations have consistently increased, whereas deceased organ donations have remained relatively unchanged [2,4].

Multiple factors, including lack of awareness, misconceptions, and socio-cultural influences, hinder the adoption of deceased organ donation. Studies have shown that public education and awareness programs are critical for improving donation rates and addressing the global shortage of transplantable organs. Nonetheless, significant barriers continue to persist despite these initiatives [6].

Several countries, including Spain, Austria, Belgium, and France, have implemented an opt-out model, in which all individuals are considered organ donors by default unless they explicitly opt-out. This approach has been shown to increase organ donation rates [6,7]. However, this system may not be enforced in countries with strong socio-cultural constraints, which must continue with an opt-in model, where organ donation occurs only after explicit consent, either before or after declaration of death. Deceased organ donation is highly dependent on the acceptance of brain death by both healthcare professionals and the public [8]. Lack of awareness regarding brain death significantly reduces the identification of potential donors. Cultural resistance and lack of confidence in the legal systems governing organ donation are particularly pronounced in Asian countries and must be addressed to mitigate organ shortages [9,10].

Therefore, this study aimed to evaluate and compare the awareness, attitudes, and perceptions toward deceased organ donation among medical students, allied health sciences (AHS) students, junior doctors, and senior doctors. By analyzing survey and interview data, the study provides insights into barriers to organ donation and identifies effective strategies, such as awareness campaigns and educational interventions, to enhance organ donation rates. Promoting accurate knowledge, addressing misconceptions, and fostering confidence in organ donation laws are essential steps toward improving access to life-saving transplantation.

## Materials and methods

Study design and materials

This mixed-methods, cross-sectional study incorporated quantitative and qualitative components. The quantitative component used a structured questionnaire to measure participants’ attitudes, awareness, and perceptions regarding organ donation. The qualitative component comprised in-depth interviews [11]. The study was conducted at a tertiary care university teaching hospital among medical students, AHS students, as well as junior and senior medical staff. Allied Health education spans a diverse range of non-physician healthcare disciplines, providing training in essential diagnostic, therapeutic, and technical services that are fundamental to the effectiveness and sustainability of healthcare delivery systems. Written informed consent was obtained from all participants. A total of 164 participants (45 medical students, 35 AHS students, 63 junior doctors, and 21 senior doctors) were included in the quantitative component. For the qualitative component, 2−3 participants from each category were interviewed.

Inclusion and exclusion criteria

Eligible participants were (1) medical students and AHS students; (2) junior doctors holding a recognized medical degree and in the first 1−2 years of residency or postgraduate training; and (3) senior doctors holding a recognized medical degree with ≥ 5 years of clinical experience in their specialty. Junior Doctors not currently practicing, and doctors ˂ 5 years of clinical experience, and individuals in predominantly administrative roles with minimal patient interaction were excluded.

Data collection

For the quantitative component, convenience sampling was used to ensure representation across the four participant categories. In the context of a single academic medical center of the study, this approach allowed for practical feasibility and operational accessibility. A structured questionnaire was customized, drawing on prior peer-reviewed literature addressing awareness and attitudes of organ donation [12,13]. To establish content validity, a multidisciplinary panel comprising two transplant surgeons, two transplant coordinators, a public health faculty member, and a biostatistician assessed item relevance and clarity and provided recommendations for refinement of phraseology. A pilot study was conducted with a small sample of participants, representative of the main study population, to validate the questionnaire and identify any ambiguities or inconsistencies. Based on pilot feedback, minor revisions were made to enhance question clarity and improve response accuracy. Additionally, pilot data were used to estimate the final sample size for the quantitative phase of the study. Based on the mean±SD of awareness scores observed in the pilot study, junior doctors (11.4 ± 2.3), medical students (11.6 ± 2.0), senior doctors (14.2 ± 0.75), and allied health science students (10.8 ± 1.6) and applying 5% relative precision with 95% confidence, the minimum required sample sizes were calculated as 63, 45, 4, and 35, respectively. The questionnaire link was circulated via institutional email/WhatsApp to all four groups, and non-responders were contacted with up to two standardized telephone reminders to invite participation. For the qualitative component, we employed purposive sampling to ensure heterogeneity of perspectives across professional tiers. Semi-structured interviews were conducted to explore in-depth perceptions and attitudes toward organ donation. A semi-structured interview guide was developed to maintain consistency while allowing participants to share their perspectives openly (Appendices). The study was conducted from April 20, 2025, to May 30, 2025. Ethical approval was obtained from the Ethics Committee of Amrita School of Medicine on April 16, 2025 (ECASM-AIMS-2025-182).

Data analysis

Quantitative data analysis was performed using descriptive statistics for comparisons between groups and to summarize closed-ended responses. Percentages and proportions were calculated to explore participants’ awareness and attitude. For qualitative data, thematic analysis was employed to analyze interview transcripts. Thematic coding was used to identify key themes, which were subsequently categorized into: awareness and knowledge, attitude toward organ donation, barriers to organ donation, and facilitators of organ donation. Coding and thematic analysis were performed using Atlas.ti software.

## Results

Demographics

The participants’ ages varied across groups, with medical and AHS students in their early 20s, junior doctors averaging 31.6 years, and senior doctors averaging 49.2 years. The gender distribution showed a higher percentage of female participants among medical students (62.2%) and AHS students (77.1%), whereas junior doctors (50.8%) and senior doctors (71.4%) had a higher percentage of male participants (Table 1).

**Table 1:** Demographics of the participants

Group	Mean Age Mean ± SD	Female n (%)	Male n (%)
Medical Students	21.1 ± 0.9	28 (62%)	17 (38%)
AHS Students	20.9 ± 1.4	27 (77%)	8 (23%)
Junior Doctors	31.6 ± 4.3	32 (51%)	31 (49%)
Senior Doctors	49.2 ± 7.8	6 (29%)	15 (71%)

Awareness and knowledge of organ donation

**Figure 1:**
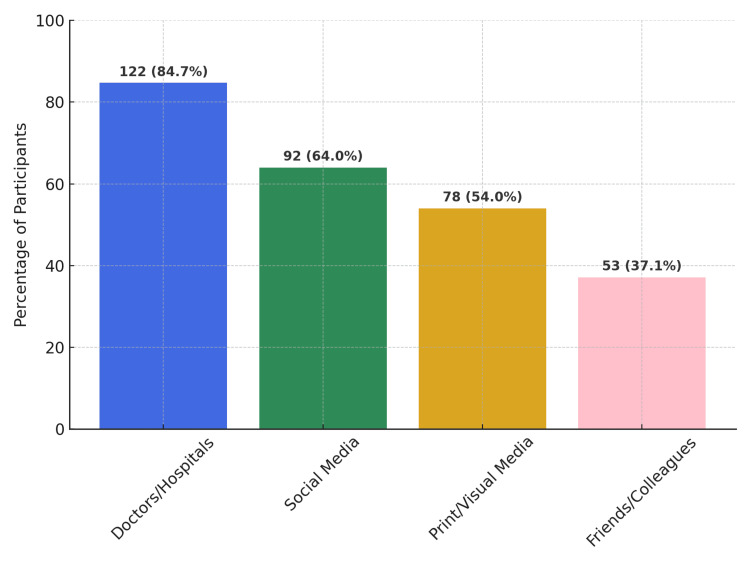
Sources of information on organ donation

Awareness of organ donation was reported by 99.3% of participants. The distribution of sources of information is illustrated in Figure 1.

All participants acknowledged that multiple organs can be donated posthumously, and 63.4% reported having a personal acquaintance with an organ donor.

**Figure 2:**
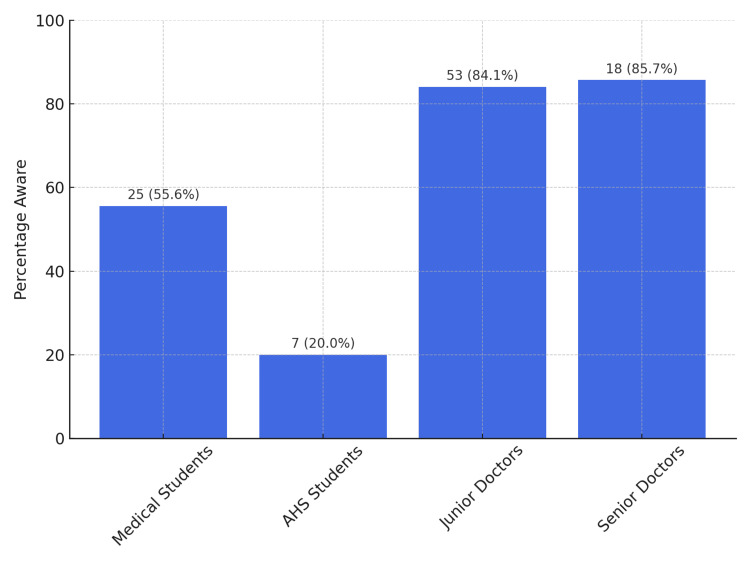
Awareness of legal framework

Awareness of the legal framework governing organ donation varied significantly across the professional tiers of this study (Figure 2).

**Figure 3:**
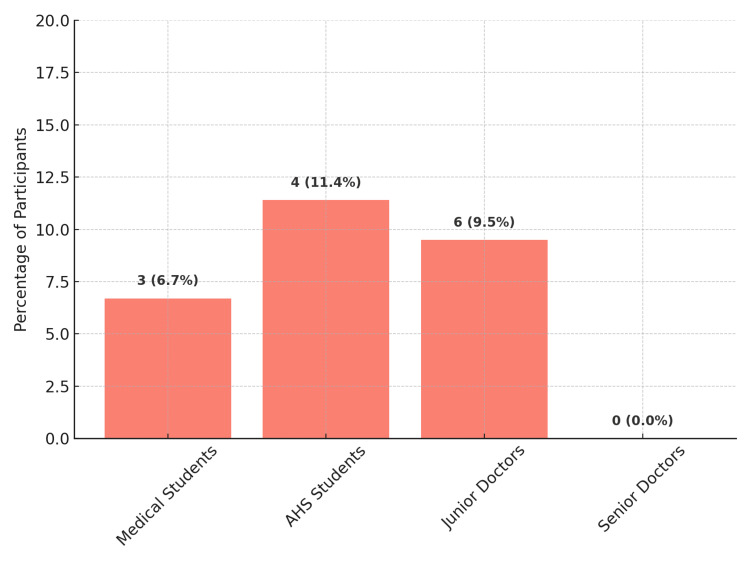
Prevalence of the misconception of brain death reversibility

In the context of brain death, 93.9% of respondents correctly identified it as the irreversible loss of cerebral function. The distribution of the misconception that brain death is reversible is presented in Figure 3. Most respondents (98.8%) agreed on the need for strict legislation to regulate organ donation.

Thematic analysis revealed gaps in awareness regarding legal policies and the concept of brain death. Some participants expressed uncertainty about brain death and its role in organ donation, with a few believing that brain-dead patients might recover. Furthermore, while senior professionals demonstrated high legal awareness, many students and junior doctors reported limited knowledge of the policies governing organ donation.

Perceptions of organ donation

**Figure 4:**
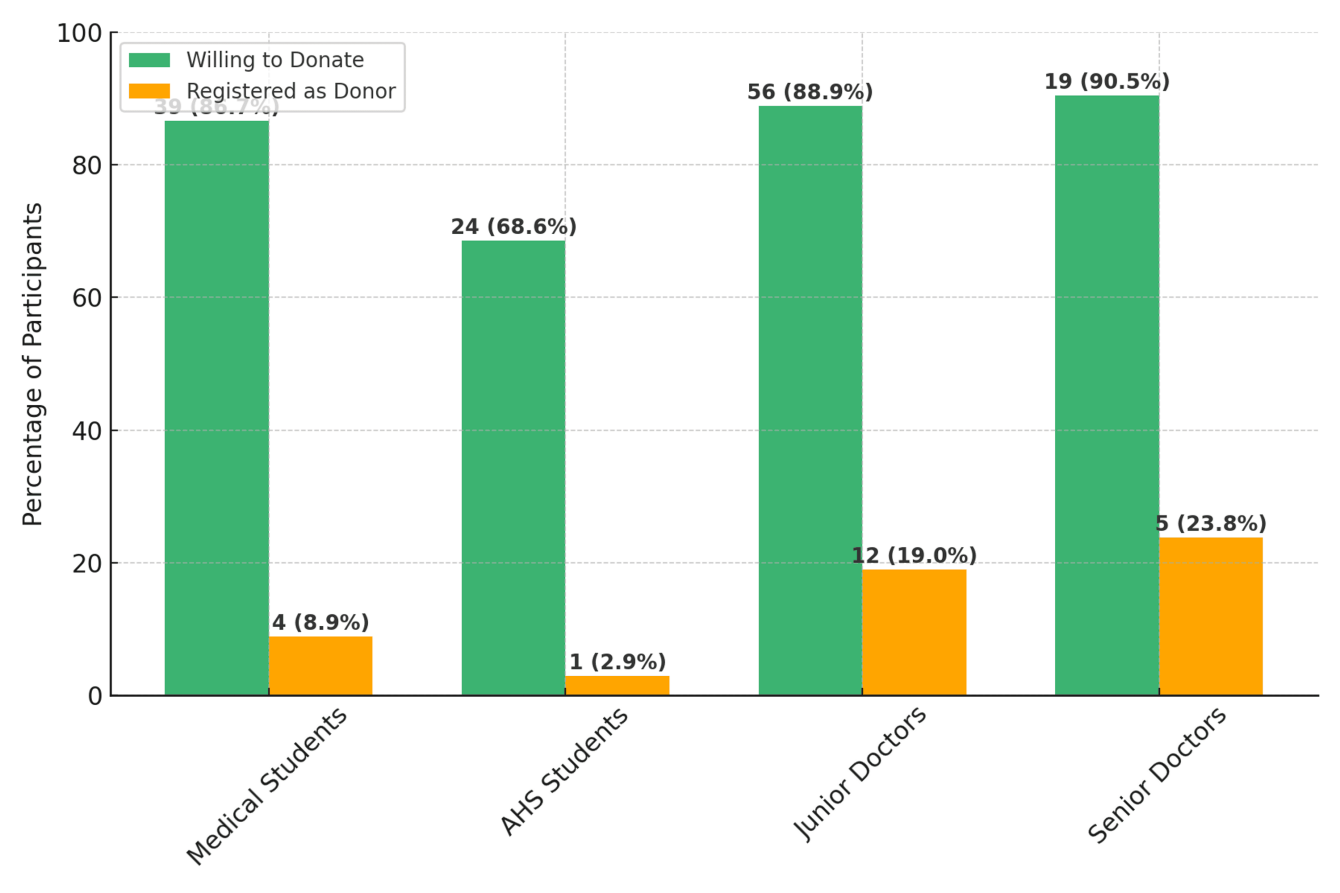
Intention-action gap - Willingness to donate organs vs. actual registration

A majority of the participants (98.2%) agreed that organ transplantation is an important intervention that improves quality of life. There was strong support for organ donation, with 84.1% willing to donate their organs after death and 86% willing to consent to organ donation by relatives. However, registration as organ donors was low, with only 13.4% of the participants possessing a donor card. Although willingness to donate after death was high, actual registrations as donors were observed to be low (Figure 4).

Despite widespread acceptance of organ donation, 17.7% of respondents indicated that their family would object to their decision to donate organs after death, and 39% were uncertain about potential familial opposition. Additionally, 44.4% of medical students and 11.4% of AHS students expressed concern that healthcare professionals might hasten the death of individuals listed as organ donors.

Interviews revealed that while willingness to donate organs was high, many participants were hesitant due to a lack of awareness about the registration process. Family influence played a major role, as some participants stated that even if they were willing to donate, their families' opinions were crucial. Ethical concerns, particularly regarding bodily integrity after death, were raised by a few participants, although most agreed that donation was a moral responsibility.

The total scores for awareness and attitude were higher among senior and junior doctors than among AHS and medical students (Table 2).

**Table 2:** Knowledge and attitude score AHS: Allied Health Sciences

Group	Mean Knowledge Score (Maximum - 16)	Mean Attitude Score (Maximum - 14)
Medical Students	13.3 ± 1.4	10.6 ± 2.3
AHS Students	12.2 ± 1.4	9.6 ± 2.3
Junior Doctors	14.8 ± 1.8	11.3 ± 2.1
Senior Doctors	15.3 ± 0.8	11.4 ± 1.9

Perceived barriers to organ donation

**Figure 5:**
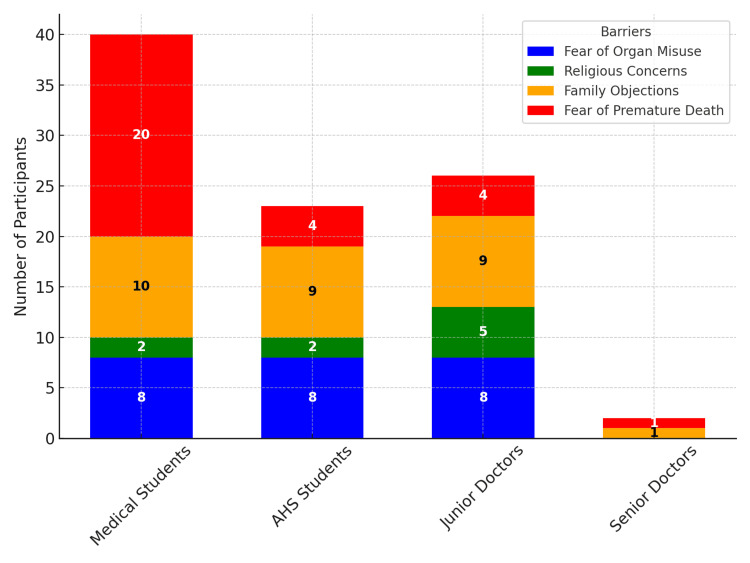
Perceived barriers to organ donation

Across participant groups, fear of premature death emerged as the most frequently cited barrier, particularly among medical students (n = 20), while it was far less common among AHS students (n = 4), junior doctors (n = 4), and senior doctors (n = 0). Family objections were another notable barrier, reported by 10 medical students, 9 AHS students, 9 junior doctors, and 1 senior doctor. Religious concerns were less common but present, especially among junior doctors (n = 5). Fear of organ misuse was reported by eight participants across all groups except senior doctors (Figure 5). Overall, the findings suggest that medical students expressed greater concerns about premature death, while experienced professionals, such as senior doctors, reported fewer perceived barriers, indicating that exposure, experience, and knowledge may reduce such apprehensions.

Among the 10 individuals selected for the qualitative phase, 8 were female participants (Table 3). Interviews revealed deep-seated concerns about organ misuse, largely influenced by media portrayals of illegal organ trade. Participants feared that their organs might not be allocated to the most deserving patients, a concern that aligns with quantitative findings. Additionally, religious beliefs contributed to hesitation, with some believing that organ donation interferes with afterlife rituals.

Facilitators and social perception

The need for government and health systems interventions was highlighted, with 98.2% of respondents supporting the implementation of promotional campaigns for organ donation. While 67.1% felt comfortable asking a relative to donate an organ, 32.9% were unwilling, reflecting a gap in acceptability and trust regarding organ transplantation.

To improve organ donation rates, participants suggested several key facilitators. They emphasized the role of physicians as educators in both clinical and community settings, encouraging proactive discussions on organ donation with patients and their families. This approach highlights the critical role healthcare professionals play in increasing awareness and understanding of organ donation. Incorporating organ donation education into medical and school curricula was proposed as a strategy to dispel myths and promote early awareness of organ donation. Participants also noted that advocacy by public figures, clear policies, and advertising campaigns could strengthen public trust and normalize discussions on organ donation.

**Table 3:** Demography of the study participants in the interview

Si. No.	Participant	Gender	Designation
1	SD01	Male	Senior Doctor
2	SD02	Female	Senior Doctor
3	SD03	Male	Senior Doctor
4	JD01	Female	Junior Doctor
5	JD02	Female	Junior Doctor
6	JD03	Female	Junior Doctor
7	MS01	Female	Medical Student
8	MS02	Female	Medical Student
9	AS01	Female	AHS Student
10	AS02	Female	AHS Student

This thematic analysis presents key themes that emerged from participant interviews, providing insights into awareness, attitudes, barriers, and facilitators for improving organ donation practices.

Awareness and knowledge

Participants demonstrated varied levels of knowledge regarding deceased organ donation. While most understood the general concept, many lacked detailed information about specific medical and legal aspects. When asked to define organ donation, participants generally described it as the process of obtaining organs from a brain-dead donor to benefit others. One participant stated, “Deceased organ donation is when organs are taken from a brain-dead person and given to someone in need” (AS01). Understanding of brain death, however, varied among participants. Some correctly explained brain death as a state in which all brain functions have ceased, while others expressed uncertainty. A junior doctor noted, “Brain death is when there is no brain activity, but other organs can still work with medical support” (JD02). Another participant highlighted its significance in organ transplantation, stating, “Brain-dead patients can no longer recover, so their organs can save others” (MS01). Knowledge of the legal framework governing organ donation was also inconsistent. While some participants were aware of the Transplantation of Human Organs Act, they lacked understanding of its provisions. One participant admitted, “I have heard about the Transplantation of Human Organs Act but do not know the details” (SD03). Another responded, “I am not sure how the legal process for organ donation works in India” (JD01). These responses suggest the need for structured educational programs that address the legal aspects of organ donation.

Attitude

Overall, participants demonstrated a positive attitude toward organ donation, with most expressing willingness to donate. However, a lack of knowledge regarding the registration process deterred many from taking action. One participant stated, “I am totally in favor of organ donation and would sign up if I knew the process” (MS02), while another participant noted, “I am interested, but I don't know where to sign up or how it works” (JD03). Family influence was considered crucial in decision-making, with most respondents indicating that their willingness to donate depended on family approval. As one participant stated, “Even if I want to donate, my family’s opinion matters; they need to be convinced too” (SD01). Another participant remarked, “I think discussing organ donation with family is crucial for making a decision” (AS03). Emotional and ethical factors also significantly influenced attitude, with concerns about bodily integrity frequently mentioned. A junior doctor stated, “It is a good cause, but the thought of my body being used after death feels uncomfortable” (JD01). Conversely, some participants framed organ donation as an ethical responsibility. One participant stated, “If my organs can save lives, I feel it is the right thing to do” (MS01), reflecting an altruistic perspective.

Barriers

Several barriers to organ donation were identified, the most prominent being a lack of awareness. Many participants were unfamiliar with the registration process, as reflected in the statement, “I don't know where to register, and no one has approached me about it” (JD02). Misconceptions about brain death also influenced attitudes, with some believing it to be reversible. One participant noted, “Some people think brain death is reversible, so they are wary” (AS01), emphasizing the need for improved medical education on the issue. Religious and cultural beliefs also affected decision-making, with concerns about maintaining bodily integrity after death, discouraging donation. An elderly medical professional stated, “Some think the body must remain whole after death for spiritual reasons” (SD03), with one participant noting, “In certain cultures, organ donation is discouraged” (JD03). Fear of organ trafficking emerged as another barrier, often fueled by portrayals of illegal organ trade activities. One participant expressed concerns, stating, “I’m afraid that organs won’t be given to those most in need but to influential people” (SD01), with another noting the media's influence in shaping such concerns, stating, “Movies and news repeatedly feature illegal organ trade, making individuals wary” (MS02). These barriers highlight the need to promote greater openness and develop strategies to enhance trust in organ donation procedures.

Facilitators

Participants highlighted several factors that could increase organ donation rates. The role of healthcare professionals as advocates was emphasized. “Doctors should take the lead in educating the public about organ donation” (AS03). Another participant highlighted the lack of proactive communication, stating, “Even healthcare professionals hardly ever mention organ donation to patients” (JD02). Education programs were also highlighted as a key strategy, with suggestions for early interventions through school curricula and medical training programs. One participant stated, “If organ donation education is introduced in schools, people will learn the right way” (MS01), while another added, “Medical students should be taught how to counsel families regarding organ donation” (SD01). Participants further emphasized the importance of the government and the media in promoting organ donation. A junior doctor stated, “If the government actively promotes organ donation, more people will have faith in the process” (JD03), and another participant endorsed the use of social media campaigns, stating, “Public figures speaking out on donation on social media would encourage more people to register” (AS01). Overall, these facilitators suggest that increasing organ donation rates requires a comprehensive approach involving education, advocacy, and policy interventions (Table 4).

**Table 4:** Representative quotes supporting findings

Theme	Sub-Theme	Code	Sample Quote
Awareness and Knowledge	Definition of Organ Donation	Understanding of deceased organ donation	“Deceased organ donation is when organs are taken from a brain-dead person and given to someone in need.” (AS01)
	Concept of Brain Death	Definition of brain death	“Brain death is when there is no brain activity, but other organs can still function with medical support.” (JD02)
		Role of brain death in organ donation	“Brain-dead patients can no longer recover, so their organs can help save others.” (MS01)
	Awareness of Laws	Familiarity with transplant laws	“I have heard about the Transplantation of Human Organs Act, but do not know the details.” (SD03)
		Lack of knowledge on policies	“I am not sure how the legal process for organ donation works in India.” (JD01)
Attitudes Toward Organ Donation	Willingness to Donate	Personal support for organ donation	“I fully support organ donation and would register if I knew the process.” (MS02)
		Hesitation due to a lack of awareness	“I am interested, but I don’t know where to register or how it works.” (JD03)
	Influence of Family	Family approval as a deciding factor	“Even if I want to donate, my family’s opinion matters. They need to be convinced too.” (SD01)
		Discussing donation with relatives	“I think talking to family about organ donation is important for decision-making.” (AS03)
	Emotional and Ethical Considerations	Concerns over bodily integrity	“It is a noble cause, but the idea of my body being used after death feels unsettling.” (JD01)
		Moral responsibility to help others	“If my organs can save lives, I feel it is the right thing to do.” (MS01)
Barriers to Organ Donation	Lack of Awareness	Limited information on donation	“I don’t know where to register, and no one has approached me about it.” (JD02)
		Misinformation about brain death	“Some people believe brain death is reversible, which makes them hesitant.” (AS01)
	Religious and Cultural Beliefs	Concerns about the afterlife and bodily integrity	“Some believe that the body should remain intact after death for spiritual reasons.” (SD03)
		Religious opposition to organ donation	“In some cultures, organ donation is not encouraged.” (JD03)
	Fear of Organ Trafficking	Concerns about illegal organ trade	“I fear that organs may not go to the most deserving but to those with influence.” (SD01)
		Media influence on organ donation fears	“Movies and news often show illegal organ trade, making people hesitant.” (MS02)
Facilitators for Organ Donation	Role of Healthcare Professionals	Doctors as advocates for donation	“Doctors should take the lead in educating the public about organ donation.” (AS03)
		Lack of proactive discussions	“Even healthcare professionals rarely discuss organ donation with patients.” (JD02)
	Educational Initiatives	Incorporating donation awareness in schools	“If organ donation awareness starts in schools, people will grow up with the right mindset.” (MS01)
		Training for medical students	“Medical students should be taught how to counsel families on organ donation.” (SD01)
	Media and Government Involvement	Government policies to promote trust	“If the government actively promotes organ donation, more people will trust the process.” (JD03)
		Social media awareness campaigns	“Public figures talking about donation on social media would encourage more people to register.” (AS01)

## Discussion

This study reveals the complex nature of awareness, attitudes, and perceptions regarding deceased organ donation among medical students, AHS, junior doctors, and senior doctors. Although there is a substantial willingness to support organ donation, gaps in knowledge, misconceptions, and various barriers continue to impede effective participation.

The mixed-methods approach enabled the examination of the intention-action gap in organ donation with greater vision and clarity by integrating quantitative findings, which establish the magnitude of the gap, with qualitative insights that reveal its underlying drivers. This approach not only strengthened the evidence base but also enhanced the policy relevance of the findings.

Awareness and knowledge of organ donation

Despite high self-reported awareness of organ donation (99.3%), qualitative findings reveal substantial knowledge gaps, particularly regarding brain death and legal policies. Some respondents incorrectly believed that brain death could be reversed, a misconception more common among AHS students and junior doctors. These findings are consistent with previous studies, which indicate that healthcare professionals are essential in promoting organ donation; however, they often lack a comprehensive understanding of brain death. For instance, a study conducted by Bharambe et al. in Pune revealed that 46.7% of final-year students believed a person can recover from brain death [14]. Similarly, Vincent et al. reported that among undergraduate medical and nursing students in southern India, only 46.9% correctly understood the concept of brain death [15]. These results emphasize the need for accurate dissemination of information regarding brain death [14,15]. Furthermore, awareness of organ donation laws varied; this underscores the importance of integrating structured education on organ transplantation laws into medical and allied health curricula, ensuring that all healthcare professionals possess accurate knowledge of the legal and ethical aspects of organ donation and transplantation.

Attitudes toward organ donation and family influence

The study found that a large majority of participants were willing to donate organs, with 84.1% indicating willingness to donate their own organs and 86% willing to consent to a loved one’s donation. A study by Vinay et al. among medical students reported that only 9.3% had a negative attitude toward organ donation, highlighting the importance of fostering a positive perception of organ donation [16]. Conversely, in a study conducted in Rwanda by Uwingabiye et al., 63% participants demonstrated a negative attitude toward kidney donation [17]. However, the actual rate of donor registration was low (13.4%), indicating a gap between intention and action, often linked to uncertainty about the registration process and the absence of active initiatives. Thematic analysis revealed that family influence is a significant determinant of decision-making, with many participants noting that their families’ support would be crucial in their choice to donate. One participant remarked, “Even if I want to donate, my family’s opinion matters; they need to be convinced too.” This finding reinforces the significance of family-focused awareness programs in fostering discussions about organ donation within households.

Barriers to organ donation

Several barriers were identified that hinder widespread participation in deceased organ donation. A primary factor was the lack of awareness of the registration process, with some participants indicating that they were unaware of how and where to register as donors.

Religious and cultural beliefs were also mentioned as barriers, with some respondents expressing the need to maintain body integrity after death for spiritual reasons. Although religious objections were low (5.4%), 22.9% of AHS students expressed uncertainty about their religious views on organ donation, highlighting the importance of engaging with religious leaders to address these concerns. Almohsen et al. reported that misconceptions regarding religion contributed to lower organ donation rates in central Saudi Arabia [18]. In contrast, a national-level survey by Chandrasekaran et al. found that only 2.5% cited religious constraints as a barrier to organ donation [19]. Kumar and Jothula in Telangana reported that 27.2% believed religious misconceptions limited acceptance toward organ donation [20].

Furthermore, fears regarding organ trafficking and misuse were common, often influenced by media portrayals of illegal organ trade. In the study by Kumar and Jothula, 45.4% of participants expressed concerns about organ misuse [20]. A participant of the present study remarked, "I fear that organs may not go to the most deserving but to those with influence." Addressing these concerns through transparent organ allocation policies and adherence to ethical guidelines is essential to foster public trust.

Facilitators and strategies to enhance organ donation

The study identified several factors that could improve organ donation rates. Healthcare professionals were recognized as key influencers, although participants noted that discussions about organ donation rarely occur in clinical settings. This finding highlights the importance of training programs that equip doctors and allied health professionals to educate patients and their families about organ donation. A comparison study by Naidoo et al. revealed that medical students demonstrated a higher willingness to donate organs than non-medical students, attributed to their greater awareness of the process and need for organ donation [21]. Educational initiatives were also highlighted as critical. Participants recommended integrating organ donation awareness into school curricula. One participant remarked, “If organ donation awareness begins in schools, people will develop the right mindset.” A study conducted among students at Topiwala National Medical College and B.Y.L. Nair Charitable Hospital in Mumbai found that 89.7% participants supported the inclusion of organ donation awareness programs in the curriculum [22]. Higher knowledge, attitude, and practice scores among MBBS students compared with students in other programs were attributed to continued medical education (CMEs) and similar training programs [22]. Furthermore, government involvement and media campaigns were identified as essential for increasing donation rates. Participants emphasized that policy-driven efforts, including mandatory organ donation education and public awareness campaigns through social media and traditional media, could dispel misconceptions and reduce fears.

Implications for policy and practice

The study has several implications for policies and practices aimed at increasing deceased organ donation rates. First, it is essential to incorporate standardized training on brain death and the legal aspects of organ donation into the curricula of medical and AHS. Prior research supports the role of positive attitudes in motivating individuals and their families to participate in organ donation (Deshpande et al. [23]; Schaffner et al. [24]). Second, public awareness campaigns should prioritize correcting misconceptions and educating families about the critical role of consent, as emphasized by several authors like Chandrasekaran et al. [19], Kumar and Jothula [20], and Vincent et al. [15]. Third, hospitals should integrate structured discussions on organ donation into end-of-life care protocols, thereby ensuring that families receive accurate information from trusted healthcare professionals. Finally, government-led initiatives to simplify donor registration and enhance transparency in organ allocation are crucial to address public concerns and encourage greater participation. For example, Kumar and Jothula reported that only 3.9% of participants were aware of the “Jeevandan” organ transplantation scheme implemented by the State of Andhra Pradesh prior to 2014 [20]. This finding stresses the importance of government initiatives and organized schemes, as recommended in the Madrid Resolution, to ensure that organ donation programs are self-sustaining, transparent, and widely accessible [4,5,20].

In reality, medical and ethical guidelines ensure that patient care is never compromised by donor status. Nonetheless, public mistrust persists as a major barrier to organ donation, with lingering fears that donor registration may reduce efforts to preserve life in critical situations. Transparent communication and targeted awareness initiatives are therefore essential to dispel such misconceptions and strengthen trust in the healthcare system. In this context, the Competency-Based Medical Curriculum (CBME) has incorporated the AETCOM module (Attitude, Ethics, and Communication), offering a structured platform to address such challenges. The urgent priority is to emphasize its practical significance, ensuring that it is not perceived as a theoretical or ornamental component. Innovative and effective strategies to integrate AETCOM across all phases of medical education are critical for preparing future healthcare professionals to address sensitive issues, such as organ donation, with both competence and compassion.

The failure to translate intention into action, referred to in the social sciences parlance as the intention-action gap, is particularly evident in the context of organ donation. Although public willingness to donate is widespread, actual donor registration and communication of consent remain comparatively low. Contributing factors include psychological barriers, cultural and religious hesitations, mistrust of healthcare, family influence, limited awareness of registration procedures, and inadequate policy support. Narrowing this gap requires both intrinsic and extrinsic motivation. Addressing these challenges through simplified registration processes, targeted public education, and community engagement is essential to convert willingness into tangible commitment. As a call to action, the famous Nike tagline ‘Just do it’ may be invoked to emphasize the importance of transforming inclination into decisive action at the grassroots level.

Limitations

This study has some limitations. First, the use of convenience sampling in the quantitative phase may limit the generalizability of the findings beyond the study setting. Second, the sample size for qualitative interviews was small, including only two to three participants from each group, which may not fully capture the range of perspectives across professional categories. Despite the small qualitative sample, themes were consistent across groups, suggesting practical saturation for core domains. Additionally, the self-reported nature of survey responses introduces the potential for social desirability bias, particularly regarding questions about willingness to donate. The study was conducted at a single tertiary care teaching hospital, which may not be representative of the broader healthcare context in India. Despite these limitations, the study provides valuable insights into awareness and attitudes toward organ donation among current and future healthcare professionals.

## Conclusions

Although awareness of organ donation was high, substantial knowledge gaps persisted regarding brain death, legal frameworks, and registration processes, contributing to a marked discrepancy between willingness to donate and actual donor registration. Family influence emerged as a critical factor in decision-making, with many participants indicating that their decisions were influenced by familial approval. Cultural beliefs and misconceptions, particularly regarding organ trafficking, were identified as key barriers, underscoring the need for transparent policies and ethical guidelines to build public trust. To improve donation rates, healthcare professionals should actively advocate for organ donation through patient education and structured discussions. Integrating formal education on brain death and legal policies into medical and allied health curricula, combined with targeted public awareness campaigns via social media and community outreach, can help normalize organ donation. A comprehensive strategy involving education, policy reform, and community engagement is essential to address these challenges, promote informed decision-making, and ultimately increase donor registration, thereby saving more lives through transplantation.

## Appendices

Questionnaire

Demographics

·      Age

·      Sex

·      Study Group: Medical Student/AHS student/Junior Doctor/Senior Doctor

**Table 5:** Knowledge

Question	Answer	Score
Have you ever heard of the term “Organ Donation”?	Yes	1
	No	0
You heard about organ donation through which of the following sources where are the options	Social Media	-
	Print and Visual Media	-
	Doctor/Hospital	-
	Friend/Colleague	-
What organs can be donated after death?	Heart	0
	Liver	0
	Pancreas	0
	Kidney	0
	Lung	0
	All of the above	1
Do you know of anyone who has donated an organ?	Yes	-
	No	-
Brain death occurs when the brain stops functioning, even if the heart is kept beating by artificial means	True	1
	False	0
Do you believe that a patient with brain death might come back to life?	Yes	0
	No	1
	No idea	0
For donation after death, who should give consent to donate organs	Spouse/family	1
	Friends	0
	Consent not required	0
Organ donors may range in age from newborn to 70 years	True	1
	False	0
Are you aware of any local or international legislation with regards to organ donation?	Yes	1
	No	0
Is there any need for having effective laws to govern the process of organ donation?	Yes	1
	No	0
When an organ is removed, the family of the deceased donor pays for the surgery to remove the organ	True	0
	False	1
Organ transplants are successful in prolonging and improving the quality of a recipient’s live	True	1
	False	0
Organ transplantation is an acceptable form of medical treatment	Agree	2
	Don’t know	1
	Disagree	0
If I register as an organ donor, I am concerned that healthcare professionals might not make full efforts to save my life in a critical situation.	Agree	0
	Don’t know	1
	Disagree	2
The donated organ may get misused	Agree	0
	Don’t know	1
	Disagree	2

**Table 6:** Attitude

Question	Answer	Score
Organ donation is a gift of life to another	Agree	1
	Don’t know	0
	Disagree	0
Organ transplants are important to help others who are very ill.	Agree	1
	Don’t know	0
	Disagree	0
If I donate my organs or tissues at the time of my death, I could be doing something good for someone else	Agree	1
	Don’t know	0
	Disagree	0
My family’s grief will somehow be reduced if my organs or tissues are donated after I die	Agree	2
	Don’t know	1
	Disagree	0
Organ and tissue donations are against my religion.	Agree	0
	Don’t know	0
	Disagree	1
Members of my family would object to donating my organs or tissues after I die	Agree	0
	Don’t know	1
	Disagree	2
Are you willing to donate your organs after your life?	Yes	1
	No	0
Have you registered for organ donation after your death / do you possess a donor card?	Yes	1
	No	0
Would you give consent to your loved one to donate their organ?	Yes	1
	No	0
Should organ donation be promoted?	Yes	1
	No	0
I would feel comfortable requesting an organ (kidney, heart, lung, liver or pancreas) donation from a family.	Yes	1
	No	0
Organ donation is a positive option for the family at the time of death of a family member	Yes	1
	No	0

Interview Guide

Introduction

Thank the participant for their time.

Explain the purpose of the interview: "This study aims to explore your knowledge, attitudes, and perceptions towards deceased organ donation, including potential barriers and facilitators."

Assure confidentiality: "Your responses will remain anonymous, and you are free to skip any question or stop the interview at any time."

Obtain informed consent before starting the interview.

Section 1: Participant Background

Can you briefly introduce yourself (role, specialty, experience in healthcare)?

Have you received any formal training or education on organ donation? If yes, what did it cover?

How often do you encounter discussions or cases related to organ donation in your field?

Section 2: Awareness and Knowledge About Organ Donation

What do you understand by the term "deceased organ donation"?

Can you explain the concept of brain death and its role in organ donation?

Are you aware of the laws and policies governing organ donation in India? (e.g., Transplantation of Human Organs Act)

Section 3: Attitudes Towards Deceased Organ Donation

How do you personally feel about organ donation? Would you consider donating your organs after death? Why or why not?

Have you signed up for an organ donor card or registry? If not, what are your reasons?

Do you think discussing organ donation with family members is important? Why or why not?

Section 4: Perceived Barriers and Challenges

What do you think are the main reasons why people hesitate to donate organs after death?

·       Lack of awareness

·       Religious/cultural beliefs

·       Myths and misconceptions

·       Fear of organ trafficking or misuse

·       Family opposition

·       Lack of trust in the healthcare system

Have you ever encountered a situation where a family refused to donate a deceased loved one's organs? What do you think influenced their decision?

In your opinion, are there enough hospital protocols and systems in place to facilitate organ donation? If not, what needs improvement?

Section 5: Facilitators and Solutions

What strategies do you think would encourage more people to register as organ donors?

·       Awareness campaigns

·       Media involvement

·       Medical training and workshops

·       Government incentives

·       Integration into medical education

Do you believe that healthcare professionals should actively advocate for organ donation? Why or why not?

How do you think the involvement of senior doctors and hospital leadership can influence organ donation rates?

17. What role do you think media and social platforms play in shaping public perceptions of organ donation?

18. Are there any specific policy changes or educational initiatives you think would be effective in increasing organ donation rates?

19. Is there anything else you would like to add about your views on deceased organ donation?

Closing

·       Thank the participant for their valuable insights.

·       Reiterate confidentiality and how the findings will be used.

Ask if they would like a summary of the study results once completed.
